# Alteration of *N*^6^ -Methyladenosine mRNA Methylation in a Rat Model of Cerebral Ischemia–Reperfusion Injury

**DOI:** 10.3389/fnins.2021.605654

**Published:** 2021-03-16

**Authors:** Dazhuang Yi, Qunhui Wang, Yuhao Zhao, Yu Song, Hong You, Jian Wang, Renjie Liu, Zhongqiang Shi, Xuan Chen, Qi Luo

**Affiliations:** ^1^Department of Neurosurgery, The First Hospital of Jilin University, Changchun, China; ^2^Department of Neurosurgery, Shanghai Tenth People’s Hospital, Tongji University School of Medicine, Shanghai, China

**Keywords:** cerebral ischemia reperfusion injury, MCAO/R, *N*^6^-methyladenosine, FTO, key genes

## Abstract

**Aim:**

This study was conducted in order to reveal the alterations in the *N*^6^-methyladenosine (m6A) modification profile of cerebral ischemia–reperfusion injury model rats.

**Materials and Methods:**

Rats were used to establish the middle cerebral artery occlusion and reperfusion (MCAO/R) model. MeRIP-seq and RNA-seq were performed to identify differences in m6A methylation and gene expression. The expression of m6A methylation regulators was analyzed in three datasets and detected by quantitative real-time polymerase chain reaction, western blot, and immunofluorescence.

**Results:**

We identified 1,160 differentially expressed genes with hypermethylated or hypomethylated m6A modifications. The differentially expressed genes with hypermethylated m6A modifications were involved in the pathways associated with inflammation, while hypomethylated differentially expressed genes were related to neurons and nerve synapses. Among the m6A regulators, FTO was specifically localized in neurons and significantly downregulated after MCAO/R.

**Conclusion:**

Our study provided an m6A transcriptome-wide map of the MACO/R rat samples, which might provide new insights into the mechanisms of cerebral ischemia–reperfusion injury.

## Introduction

As one of the most damaging neurological diseases, ischemic stroke (IS) accounts for approximately 80% of all strokes with a high risk of mortality and disability and affects approximately three million people in China (Jiang et al., 2016; Dabrowska-Bender et al., 2017; Wang et al., 2017). Ischemic stroke is caused by a sudden disruption of blood flow to the brain, resulting in brain cell death and neurological deficits (Zhang et al., 2012). At present, intravenous thrombolysis and mechanical thrombectomy are approved therapies for the acute treatment of cerebral IS (Powers et al., 2019). However, cerebral ischemia–reperfusion injury (CIRI), a common complication of IS, frequently occurs during thrombolytic or surgical treatments (Xu et al., 2018). CIRI usually causes malignant cerebral edema, blood–brain barrier disruption, and neuronal apoptosis (Liu et al., 2020). Over the past 40 years, basic experiments have revealed that the molecular mechanisms of CIRI involve multiple pathological processes, such as oxidative stress, apoptosis, inflammation, autophagy, and necrosis (Meng et al., 2018; Zhang et al., 2019). However, the neuroprotective strategies based on these mechanisms in clinical experiments have not yet provided the expected clinical outcomes (Barer and Berge, 2017), suggesting that there are other mechanisms by which CIRI affects the prognosis of patients with IS.

*N*^6^-methyladenosine (m6A), the most common and abundant RNA molecular modification in eukaryotes, governs the fate and function of the transcripts *via* an m6A-specific modulator (Li et al., 2019; Shafik et al., 2020). Recent findings indicate that the m6A modification is reversible and dynamically regulated by “writer” methyltransferases (*Mettl3*, *Mettl14*, *WTAP*, *Zc3h13*, *Rbm15/15b*) that catalyze the addition of m6A (Agarwala et al., 2012; Horiuchi et al., 2013; Patil et al., 2016; Chai et al., 2019), “eraser” demethylases (*FTO* and *Alkbh5*) that catalyze the removal of m6A (Rajecka et al., 2019), and “reader” proteins (*Ythdc1*, *Ythdc2*, *Ythdf1*, *Ythdf2*, *Ythdf3*, *Hnrnpc*, *Hnrnpa2b1*, *Eif3a*, and *Eif3c*) that specifically recognize it (Wang X. et al., 2015; Li et al., 2019). Many studies have revealed that m6A modification regulates a variety of RNA metabolic processes, such as mRNA stability (Chen et al., 2018), splicing (Bartosovic et al., 2017), nuclear transport, and translation ability (Meyer et al., 2015). The knockdown of *METTL3* or *YTHDF2 in vitro* was found to increase the stability and expression of peroxisome proliferator activator receptor α (*PPARα*) mRNA (Zhong et al., 2018). Although dysregulated m6A modification has been demonstrated to be involved in various types of cancer and brain diseases (Li et al., 2019; Shafik et al., 2020), the role of m6A in CIRI has not been elucidated.

**FIGURE 1:**
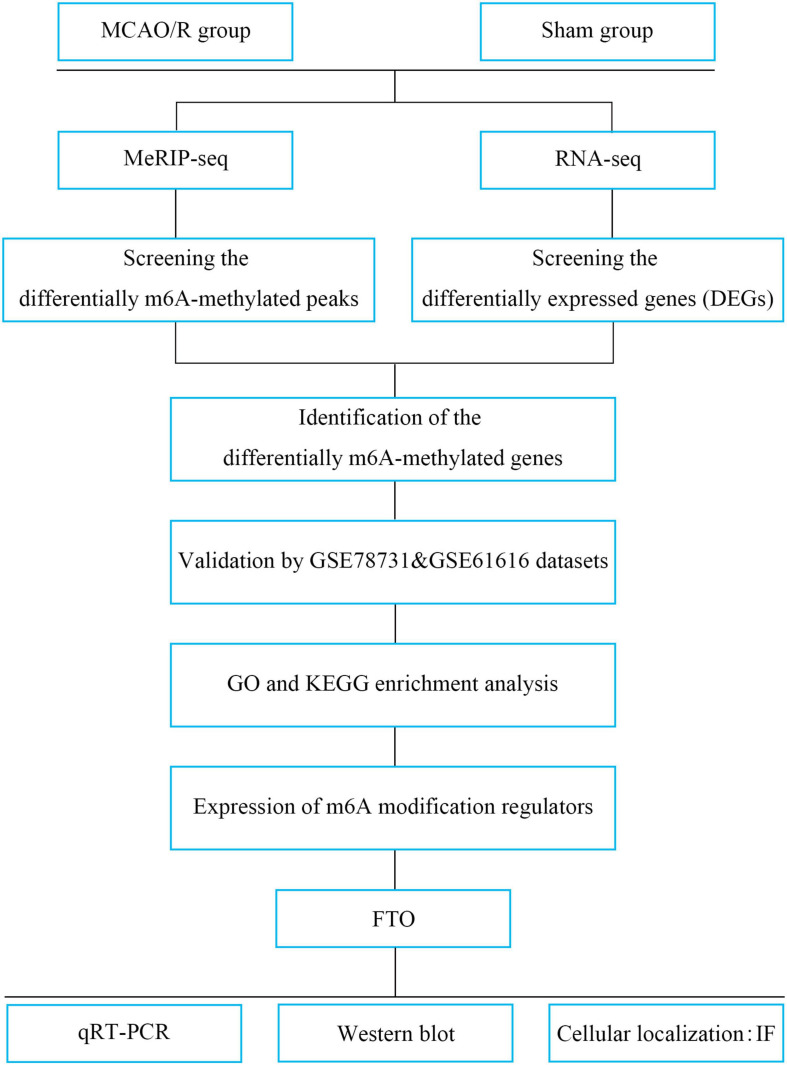
Study flowchart. MCAO/R, middle cerebral artery occlusion and reperfusion; MeRIP-seq, methylated RNA immune precipitation sequencing; DEGs, differentially expressed genes; m6A, *N*^6^-methyladenosine; GO, Gene Ontology; KEGG, Kyoto Encyclopedia of Genes and Genomes; FTO, fat mass and obesity-associated protein; qRT-PCR, quantitative real-time PCR; IF, immunofluorescence.

In the present study, we performed methylated RNA immunoprecipitation sequencing (MeRIP-seq) and RNA transcriptome sequencing (RNA-seq) to profile the changes in transcriptome-wide m6A modification sites and mRNA expression in middle cerebral artery occlusion and reperfusion (MCAO/R) rats. Additionally, two publicly available datasets were used to verify the candidate genes. We then evaluated the expression of m6A regulators (Figure 1). From the present study, it could be speculated that m6A methylation may be a breakthrough point to improve the treatment of IS.

## Materials and Methods

### Animals and Experimental Groups

All experimental protocols for this study were approved by the Animal Experimental Ethics Committee of the First Hospital of Jilin University (No. 20200707, Changchun, China). The procedures followed the International Association for the Study of Pain guidelines for animal research and standard biosecurity and institutional safety procedures. Male Sprague–Dawley rats, weighing 200–250 g, were purchased from the Experimental Animal Center of Jilin University. All rats were acclimated to a 12-h light/dark cycle with free access to food and water for 1 week. They were fasted for 12 h prior to experimentation but allowed free access to water. The rats used in this study were randomly divided into two parts: part 1 for sequencing experiments (RNA-seq and MeRIP-seq; sham and MCAO/R-24 h, *n* = 3) and part 2 for experiments such as quantification of the m6A level, quantitative real-time polymerase chain reaction (qRT-PCR), western blot, and immunofluorescence [sham (*n* = 10), MCAO/R-24 h (*n* = 10), MCAO/R-48 h (*n* = 5), and MCAO/R-72 h (*n* = 5)].

### Middle Cerebral Artery Occlusion and Reperfusion Model

**FIGURE 2:**
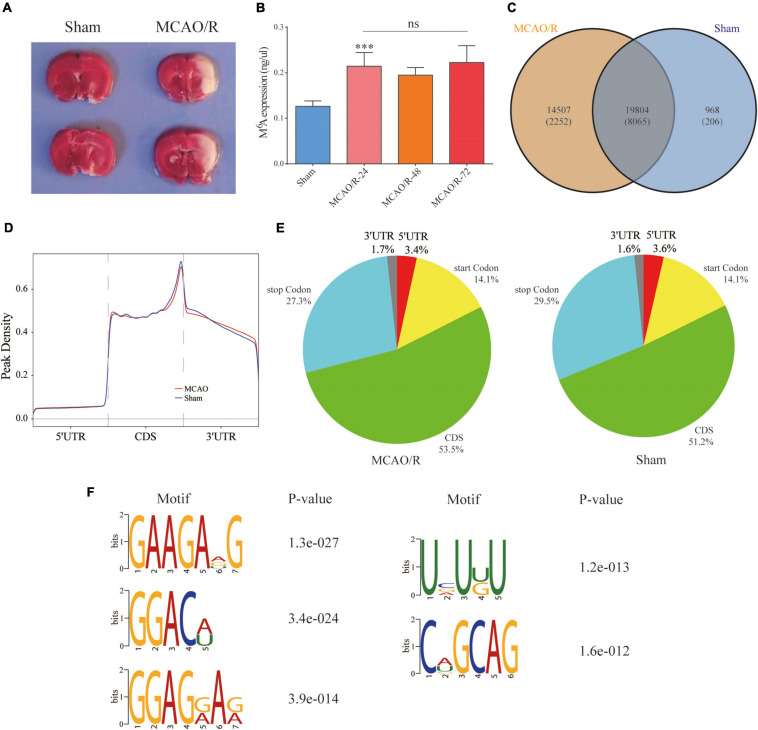
Overview of the *N*^6^-methyladenosine methylation map in the MCAO/R and sham groups. **(A)** TTC staining was used to verify the MCAO/R model 24 h after surgery. **(B)** The m6A levels of total RNA in the brain cortex were determined at the indicated time points after MCAO/R (*n* = 5). Data are presented as the mean ± SD. ***p < 0.001 and ^ns^*p* > 0.05, ANOVA with Tukey’s *post hoc* test. **(C)** Venn diagram showing the overlap of the m6A peaks within mRNAs in the two groups. **(D)** Density curve showing the distribution of m6A peaks across the transcripts. The transcript is divided into three parts, namely 5′UTR, CDS, and 3′UTR. **(E)** Pie charts showing the proportion of m6A peak distribution in the sham and MCAO/R samples. **(F)** The top five motifs enriched across the m6A peaks. TTC, 2,3,5-triphenyltetrazolium chloride; CDS, coding DNA sequence; UTR, untranslated region; SD, standard deviation.

Sprague–Dawley rats underwent the MCAO/R model of focal CIRI as previously mentioned (He et al., 2019; Gao et al., 2020). Briefly, a 0.26-mm monofilament nylon suture with a heat-rounded tip was inserted into the lumen of the right internal carotid artery through the external carotid artery until the rounded tip reached the entrance to the middle cerebral artery (MCA). The nylon suture was inserted approximately 18–20 mm (from the bifurcation of the common carotid artery) to the anterior cerebral artery, where the MCA blood flow was blocked. In the sham group, all procedures were the same as those for the MCAO/R group, except for the suture insertion. After a 90-min ischemic period, the suture was removed to restore the blood flow for reperfusion injury. The rats were anesthetized and sacrificed for different assays at the respective time points. Infarction caused by 90 min of MCAO/R was confirmed by 2,3,5-triphenyltetrazolium chloride (TTC) staining as previously described (Figure 2A; Gao et al., 2020).

### Quantification of the m6A Level in Total RNA

The m6A level in total RNA was detected using a commercial m6A RNA methylation quantification kit (P-9005, EpiGentek, United States). Briefly, 200 ng of total RNA was added into each well, and the capture antibody solution and detection antibody solution were added according to the manufacturer’s protocol. The absorbance of each well at a wavelength of 450 nm was colorimetrically measured for the m6A level.

### MeRIP-Seq and Data Analysis

Cloudseq Biotech Inc (Shanghai, China) provided the MeRIP-seq service. Briefly, m6A RNA immunoprecipitation was performed with the GenSeqTM m6A RNA IP kit (GenSeq Inc., China) according to the manufacturer’s instructions. Both the input sample without immunoprecipitation and the m6A IP samples were used for RNA-seq library generation with NEBNext^®^ Ultra II Directional RNA Library Prep Kit (New England Biolabs, Inc., United States). The library quality was evaluated using the BioAnalyzer 2100 system (Agilent Technologies, Inc., United States). Library sequencing was performed on an Illumina HiSeq instrument with 150 bp paired-end reads.

Paired-end reads were harvested from an Illumina HiSeq 4000 sequencer and were quality controlled by Q30. Then, 3′ adaptor-trimming and low-quality reads were removed using the Cutadapt software (v 1.9.3). The clean reads of all libraries were aligned to the reference genome (UCSC RN5) using Hisat2 software (v 2.0.4). Methylated sites on RNA (peaks) were identified using MACS software. Differentially methylated sites (fold changes ≥ 2 and *p*-value < 0.05) were identified by diffReps (Shen et al., 2013). These peaks identified by both software overlapping with exons of mRNA were determined and chosen by in-house scripts.

### RNA-Seq and Data Analysis

Total RNA from the brain cortex was isolated using TRIzol reagent (Life Technologies). The quality and quantity of RNA were assessed using a NanoDrop ND-1000 (NanoDrop). Denaturing agarose gel electrophoresis was used to assess the RNA integrity. RNA libraries were prepared using a NEBNext^®^ Ultra II Directional RNA Library Prep Kit (New England Biolabs, Inc., United States) according to the manufacturer’s protocol. Paired-end reads were harvested from an Illumina NovaSeq 6000 sequencer and were quality controlled by Q30. Then, 3′ adaptor-trimming and low-quality reads were removed using the Cutadapt software (v 1.9.3), and the high-quality trimmed reads were aligned to the rat reference genome (UCSC RN5). Then, the Cuffdiff software (v 2.2.1, part of Cufflinks) was used to obtain the gene level FPKM as the expression profiles of mRNA and differentially expressed mRNAs were identified (fold changes ≥ 2 and *p*-value < 0.05).

### Public Datasets and Analysis

GSE78731 (Oh et al., 2018) and GSE61616 (Wang L. et al., 2015) derived from the Gene Expression Omnibus database^1^ were explored to test the expression of differentially methylated genes between the sham and MCAO/R groups. The details of the datasets are shown in Table 1. The limma (Ritchie et al., 2015) package was used to identify differentially expressed genes (DEGs) with a *p*-value of <0.05 and an absolute fold change (FC) value of ≥2. We then screened the common DEGs in both the GSE163614 and GSE78731 (or GSE61616) datasets for the Gene Ontology (GO) and Kyoto Encyclopedia of Genes and Genomes (KEGG) pathway enrichment analyses, which were performed using the clusterProfiler (Yu et al., 2012).

**TABLE 1:** Detailed information about the datasets.

Datasets	Platform		MCAO/R group	Sham group	References
GSE61616	GPL1355	[Rat230_2] Affymetrix Rat Genome 230 2.0 Array	5	5	Wang X. et al. (2015)
GSE78731	GPL15084	Agilent-028279 SurePrint G3 Rat GE 8x60K Microarray	5	5	Oh et al. (2018)
GSE163614	GPL25947	Illumina NovaSeq 6000 (*Rattus norvegicus*)	3	3	–

We reviewed the published literature and collated a list of 17 m6A RNA methylation regulators, including six writers, two erasers, and nine readers (Chai et al., 2019; Li et al., 2019). Then, we restricted the list to genes with available RNA expression data in the three datasets and systematically compared the expression of these m6A methylation regulators in the MCAO/R and sham groups. Then, the pheatmap package in R (v 3.4.2) was used to cluster the m6A regulators.

### Quantitative Real-Time PCR

The brain cortex was harvested 24 h after MCAO/R for quantitative real-time PCR (qRT-PCR). Tissue samples were homogenized in TRIzol reagent (Invitrogen, Carlsbad, CA, United States). Total cellular RNA was extracted and transcribed to cDNA using the PrimeScript RT Reagent kit (Thermo Fisher Scientific). The primers were synthesized by Sangon Biotech (Shanghai, China; Table 2 and Supplementary Table 1). Quantitative PCR was performed using the Hieff^TM^ qPCR SYBR Green Master Mix (Low Rox Plus) (Yisheng Biotech, Shanghai, China) on the MxPro-Mx3005 P Real-time PCR system (Agilent Technologies, United States). The mRNA expression levels were calculated using the 2-ΔΔCt method and normalized to the quantity of glyceraldehyde 3-phosphate dehydrogenase (GAPDH) mRNA.

**TABLE 2:** Primers used in the present study for qRT-PCR.

Gene	Direction	Sequence (5′–3′)	Product size (bp)
*FTO*	Forward	TTCTGTCTGCCGTCCTGATCCC	113
	Reverse	GTCTGGTTTCTGCTGTGCTGGTAG	113
*Alkbh5*	Forward	GGAAGTACCAGGAGGACTCAGACC	96
	Reverse	GGATGCCGCTCTTCACCTTGC	96
*Ythdf2*	Forward	TTGCCTCCACCTCCACCACAG	111
	Reverse	CCCATTATGACCGAACCCACTGC	111
*Hnrnpa2b1*	Forward	CCAGGACCAGGAAGCAACTTTAGG	134
	Reverse	CCTCCTCCATAACCAGGGCTACC	134
*Eif3a*	Forward	CCGTCCTTCCTGGCGTAATGC	100
	Reverse	GGGTGGTCTGTCATCATCTGTGTG	100
*Eif3c*	Forward	AAGACAACATCCAGCACGCAGAC	150
	Reverse	GCTCCTTGGCACGACCACTTG	150
*GAPDH*	Forward	GACAACTTTGGCATCGTGGA	133
	Reverse	ATGCAGGGATGATGTTCTGG	133

### Western Blot

The brain cortex was harvested 24 h after MCAO/R for western blotting. Brain tissue was homogenized in RIPA buffer (0.1 g/ml) (P0013B, Beyotime, Shanghai, China) at 4°C. The protein concentration was measured using BCA assay (P0010, Beyotime). Equal amounts of protein were loaded in the sulfate-polyacrylamide gel electrophoresis (SDS-PAGE) gel, and the following antibodies were employed: anti-fat mass and obesity-associated protein (FTO) (1:1,000, abclonal, Wuhan, China), anti-GAPDH [1:1,000, Cell Signaling Technology (CST), MA, United States] and HRP-conjugated secondary antibody (1:3,000, CST). Protein blots were visualized with a chemiluminescence detection kit (NCI5079, Thermo Fisher Scientific, Waltham, MA, United States). The data were analyzed using ImageJ software (v 1.52r, Bethesda, MD, United States).

### Immunofluorescence Staining

Rats were anesthetized and successively perfused with 200 ml of 4°C normal saline and 100 ml of 4°C 4% paraformaldehyde 24 h after the MCAO/R surgery. The brains were quickly collected for paraffin embedding. Immunofluorescence staining was performed on paraffin sections (3 μm). Sections were incubated with primary antibodies against FTO (1:200, abclonal), NeuN (1:100, Abcam, Cambridge, United Kingdom), Iba1 (1:50, Santa Cruz, Dallas, United States), and GFAP (1:100, Proteintech, Wuhan, China) for 1 h at room temperature, followed by Alexa Fluor 555 (Life Technology, United States) or FITC-labeled (Sigma, United States) secondary antibodies after the sections were washed three times with PBS. Images were captured using an Olympus BX51 TRF fluorescent/light microscope system (Olympus, Tokyo, Japan).

### Statistical Analyses

Data from all the experiments are presented as the mean ± standard deviation (SD). Differences between two groups or multiple groups were analyzed by Student’s *t*-test or one-way analysis of variance (ANOVA), respectively. Statistical analyses were performed using R software (v 3.4.2) and GraphPad Prism software (v 8.00). A *p*-value of <0.05 was considered statistically significant.

## Results

### Overview of the m6A Methylation Map in MCAO/R Rats

As shown in Figure 2B, we quantified the m6A levels in the brain cortex after MCAO/R. We found that the total m6A levels were significantly increased compared with the sham group (*p* < 0.001), but there were no significant differences between the indicated time points in the MCAO/R group. Thus, three biological duplicates from the sham and MCAO/R (24 h after the operation) groups were selected for MeRIP-seq. According to the m6A MeRIP-enriched regions (peaks), 20,772 m6A peaks within 8,271 coding gene transcripts (mRNAs) were identified in the sham group. In the MCAO/R group, there were 34,311 m6A peaks within 10,317 mRNAs. Of these, 19,804 peaks within 8,065 mRNAs (56.1% of all peaks in the sham and MCAO/R groups) overlapped (Figure 2C), which confirmed that the brain is an organ with an abundance of m6A methylation (Meyer et al., 2012). To reveal the preferential distribution of m6A in transcripts, the metagene profiles of all identified m6A peaks were investigated in the entire transcriptome. Our results showed that m6A peaks were mainly enriched in the coding sequence (CDS), stop codon, and start codon in both groups (Figures 2D,E). To investigate whether the identified m6A peaks shared the consensus motif, the m6A methylomes of the sham and MCAO/R samples were mapped using HOMER software (Heinz et al., 2010). The results showed that m6A mainly exists in the 5-RRAGH-3 and 5-RRACH-3 (R = A or G; H = A, C, or U) consensus sequence. The top five conserved motifs among the identified m6A peaks are shown in Figure 2F, which were similar to previous studies (Meyer et al., 2012).

### Conjoint Analysis of the MeRIP-Seq and RNA-Seq Data

**FIGURE 3:**
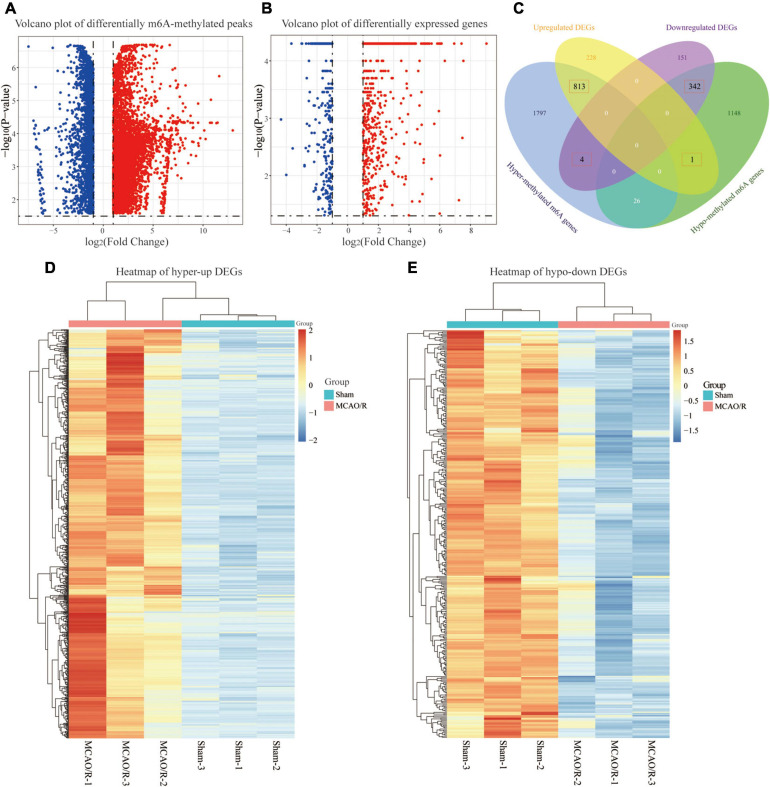
Conjoint analysis of the MeRIP-seq and RNA-seq data. **(A)** Volcano plot showing the differentially m6A-methylated peaks with fold changes ≥ 2 and *p*-value of <0.05. The red plots represent hypermethylated m6A peaks, and the blue plots represent hypomethylated m6A peaks. **(B)** Volcano plot displaying the differentially expressed genes (DEGs) with a *p*-value of <0.05 and fold changes ≥ 2. The red plots represent the upregulated genes, and the blue plots represent the downregulated genes. **(C)** Venn diagram showing the overlap of the differentially m6A-methylated genes and differentially expressed genes. **(D,E)** Heatmaps showing the expression profiles of the hyper-up DEGs **(D)** and hypo-down DEGs **(E)**. A gradual change in the color from orange to blue indicates a change in expression values from high to low.

To further elucidate the m6A methylation changes associated with CIRI, a differential m6A methylation level analysis was performed. As shown in Figure 3A, compared with the sham group, a total of 8,833 differentially methylated m6A-methylated peaks within 4,131 coding genes were identified in the MCAO/R group. Further analysis of the distribution of differentially methylated m6A peaks showed that most of them were enriched in the CDS region (Supplementary Figure 1). Among the differentially m6A-methylated peaks, 6,049 hypermethylated m6A peaks (representing 2,640 genes) were found, while 2,784 hypomethylated m6A sites (representing 1,517 genes) were discovered (fold changes ≥ 2, *p* < 0.05; Figure 3A). In addition, transcriptome profiles of altered genes were determined by RNA-seq. We identified 1,539 DEGs between the sham and MCAO/R groups, including 1,042 upregulated genes and 497 downregulated genes (fold changes ≥ 2 and *p* < 0.05; Figure 3B). Through the conjoint analysis of MeRIP-seq and RNA-seq data, we identified 817 DEGs with hypermethylated m6A peaks, of which 813 genes were significantly upregulated (hyper-up) and 4 genes were downregulated (hyper-down), and 343 DEGs had hypomethylated m6A peaks, consisting of 1 upregulated (hypo-up) gene and 342 downregulated (hypo-down) genes (Figure 2C). Based on the results above, we found that DEGs with altered m6A-methylated peaks were mainly enriched in the “hyper-up” or “hypo-down” (Figure 3C), indicating that m6A modifications tend to have a positive correlation with mRNA expression in CIRI. Hierarchical clustering was performed to show the distinguishable expression patterns of hyper-up or hypo-down DEGs between the sham and MCAO/R groups (Figures 3D,E).

### Analysis of the GSE78731 and GSE61616 Datasets and Identification of Key Genes

**FIGURE 4:**
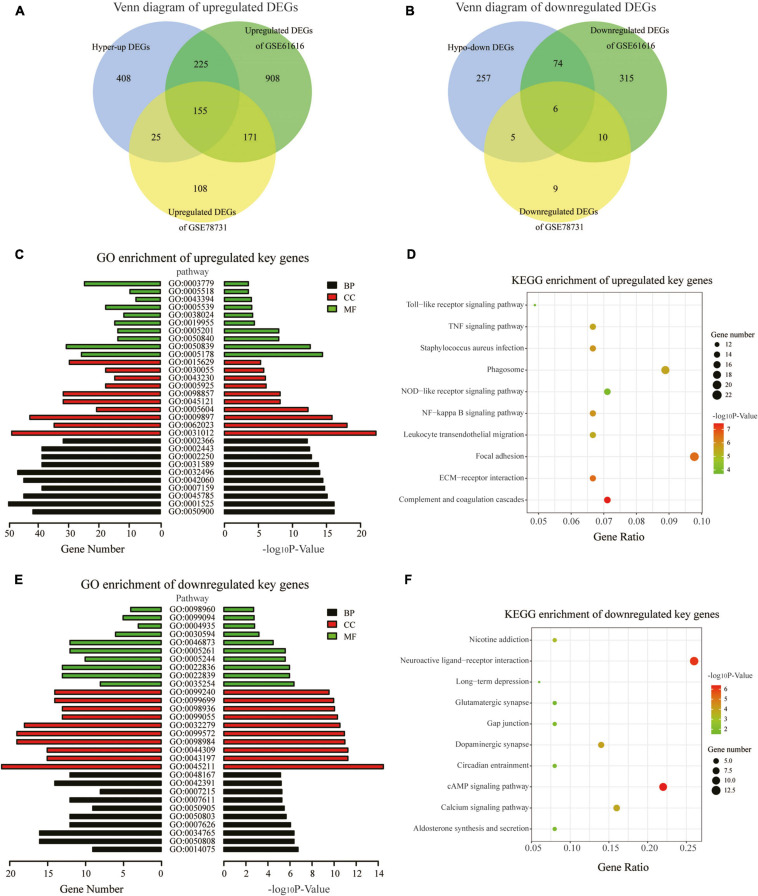
GO and KEGG pathway analyses of the key genes. **(A,B)** Venn diagrams displaying the overlap of the upregulated DEGs **(A)** and downregulated DEGs in the three datasets **(B)**. **(C)** GO annotations of the upregulated key genes with the top 30 terms. **(D)** KEGG pathway enrichment analysis of the upregulated key genes with the top 10 terms. **(E)** GO annotations of the downregulated key genes with the top 30 terms. **(F)** KEGG pathway enrichment analysis of the downregulated key genes with the top 10 terms. (GO terms and KEGG pathways with the threshold *p*-values of <0.05 were considered significant).

We downloaded two rat MCAO/R datasets (GSE78731 and GSE61616) as the test sets to validate differentially methylated and synchronous DEGs. After data preprocessing, the normalized data represented the distribution from sample to sample, as shown in the box plots (Supplementary Figures 2A,C), indicating the data’s reliability. Four hundred and eighty-nine genes were differentially expressed, namely 459 upregulated genes and 30 downregulated genes in GSE78731 (Supplementary Figure 2D). As for GSE61616, we screened 1,865 DEGs consisting of 1,460 upregulated genes and 405 downregulated genes (Supplementary Figure 2B). We then screened out the common upregulated or downregulated DEGs in both the GSE163614 (hyper-up or hypo-down DEGs) and GSE78731 (or GSE61616) datasets. A total of 405 common upregulated genes and 85 common downregulated genes (Figures 4A,B and Supplementary Table 2) were identified and regarded as “key genes” for further analysis. In particular, there were 155 hyper-up DEGs and 6 hypo-down DEGs that were present in all three datasets (Supplementary Table 2). Among them, the expressions of the top five hyper-up and hypo-down DEGs were validated by qRT-PCR, which were consistent with our bioinformatics analysis results (Supplementary Figure 3).

### Functional Enrichment Analysis of the Key Genes

To explore the physiological and pathological significance of m6A modification after CIRI, GO and KEGG pathway analyses were performed for the identified key genes. The GO analysis revealed that the upregulated key genes were significantly associated with leukocyte and immune responses, such as leukocyte migration biological process (BP), extracellular matrix cellular component (CC), and integrin binding [molecular function (MF); Figure 4C]. The downregulated key genes were mainly involved in neuron synapses and ion channel activity, for example, synapse organization BP, postsynaptic membrane CC, and ion gated channel activity MF; Figure 4E). With threshold values of *p* < 0.05, the top 30 GO terms enriched by up-/downregulated key genes (ranged by *p*-value and category) are presented in Supplementary Tables 3, 4. KEGG pathway analyses showed that the differentially methylated key genes were mainly enriched in the complement and coagulation cascades, ECM–receptor interaction, and NF-kappa B signaling pathway (for upregulated key genes, Figure 4D) and in the cAMP signaling pathway, neuroactive ligand–receptor interaction, and dopaminergic synapse (for downregulated key genes, Figure 4F).

### Expression Level of m6A RNA Methylation Regulators in CIRI

**FIGURE 5:**
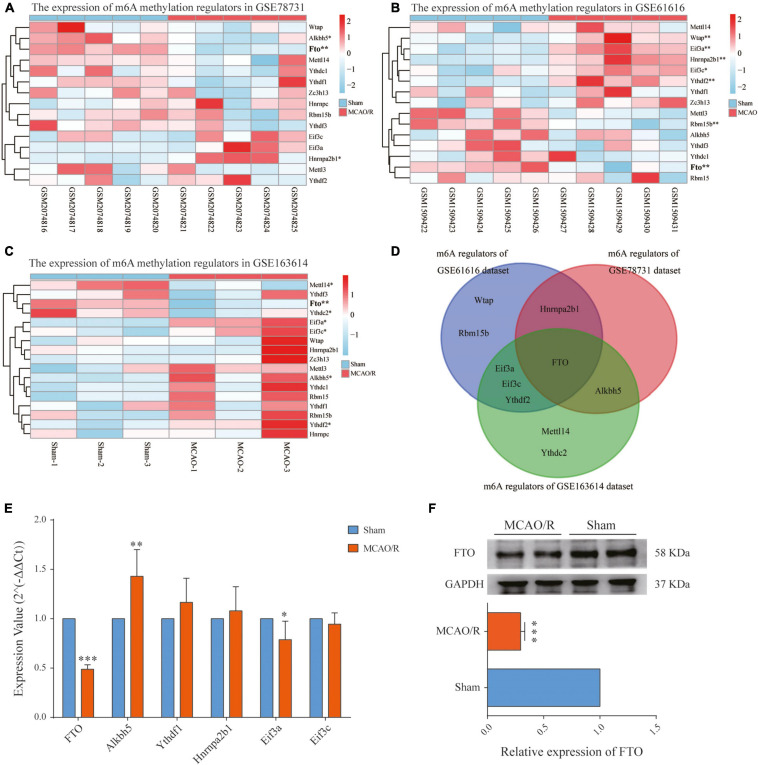
Identification of the main m6A regulator in cerebral ischemia–reperfusion injury. **(A–C)** Heatmaps showing the expression levels of m6A methylation regulators in the GSE78731 dataset **(A)**, GSE61616 dataset **(B)**, and RNA-seq data **(C)**. **(D)** Venn diagram of the m6A methylation regulators between the three datasets, of which FTO is present in all three datasets. **(E)** qRT-PCR showing the mRNA expressions of *FTO*, *Alkbh5*, *Ythdf1*, *hnrnpa2b1*, *Eif3a*, and *Eif3c* in the sham and MCAO/R groups. **(F)** FTO protein expressions determined by western blotting were significantly decreased after MCAO/R surgery. Data are presented as the mean ± SD. *p < 0.05; **p < 0.01; ***p < 0.001 vs. the sham group, *n* = 5, Student’s *t*-test.

The increased m6A level in the brain cortex after MCAO/R could be due to either the upregulation of the m6A methylase complex or the downregulation of m6A demethylases. Therefore, we investigated each m6A methylation regulator’s expression level in the three datasets as a heatmap (Figures 5A–C). Then, we selected the common regulators (FTO, Alkbh5, Ythdf1, Hnrnpa2b1, Eif3a, and Eif3c), which were present in at least two datasets for qRT-PCR validation (Figure 5D). The qPCR results showed that the FTO mRNA level was significantly decreased (*p* < 0.001), while the Alkbh5 mRNA level was significantly increased (*p* < 0.01) in the rat brain cortex after MCAO/R (Figure 5E). Combining the above results, among the six common regulators, FTO has the same downregulation expression pattern in all three datasets (*p* < 0.01), as validated by qRT-PCR and western blotting (*p* < 0.001, Figures 5E,F). Thus, FTO might be responsible for the increased m6A levels in CIRI.

### Cellular Distribution of FTO in the Brain Cortex After MCAO/R

**FIGURE 6:**
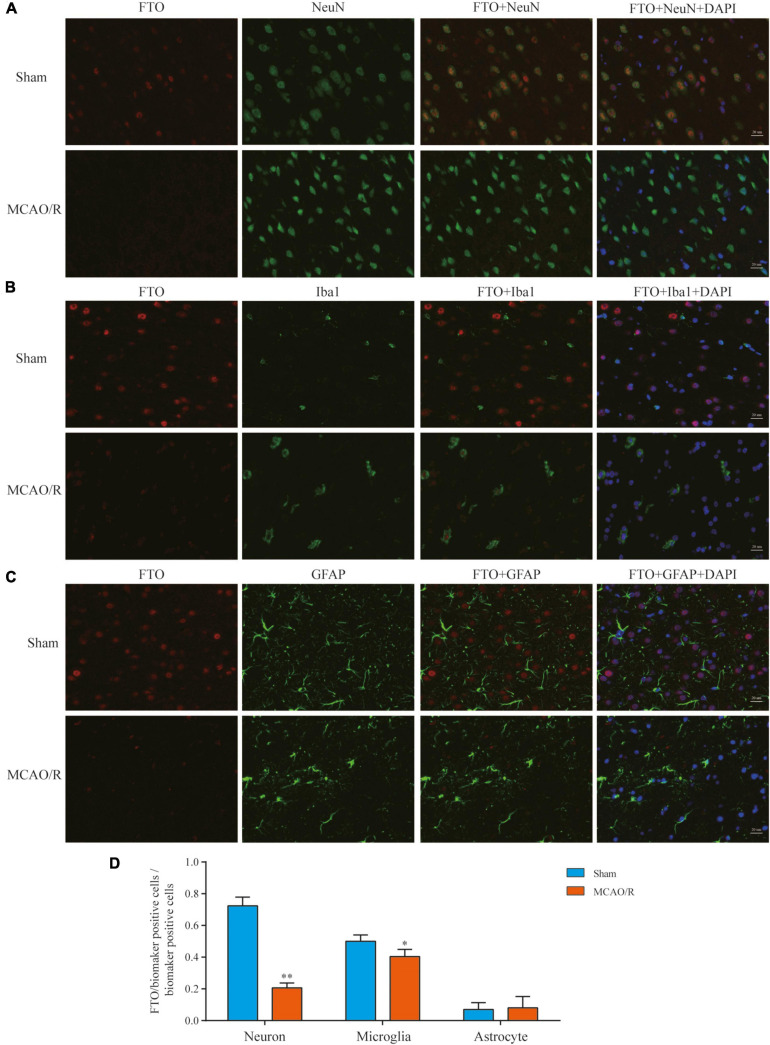
Expression and distribution of FTO detected by immunofluorescence. Representative photomicrographs of double immunofluorescence staining for FTO (red)/NeuN (green), FTO (red)/Iba1 (green), and FTO (red)/GFAP (green) in each group showed that the FTO was mainly localized and specifically downregulated in neurons **(A)** and some in microglia **(B)**. In contrast, it was rarely expressed in astrocytes **(C)** 24 h after MCAO/R. The nucleus was counterstained with 4’,6-diamidino-2-phenylindole (DAPI) (blue) in the same view in each section. Scale bar: 20 μm. **(D)** Quantification of FTO expression in the neurons/microglia/astrocytes. *p < 0.05; **p < 0.01 vs. the sham group, *n* = 5, Student’s *t*-test.

FTO is a well-known m6A demethylase that plays a critical role in m6A methylation. To further elucidate FTO’s expression and distribution, immunofluorescence staining was performed in the rat brain cortex. In the sham-operated rat brain, FTO was mainly observed in neurons (NeuN+) and some microglia (Iba1+), whereas it was rarely expressed in astrocytes (GFAP+). In the MCAO/R groups, there was almost no FTO detected in these three cell types (Figure 6). At 24 h after reperfusion following transient MCAO, neuronal FTO immunostaining decreased significantly compared with the sham group (Figures 6A,D).

## Discussion

Due to advances in sequencing technology, researchers have discovered that the brain shows higher m6A abundance than other organs in mammals (Meyer et al., 2012). Numerous studies have demonstrated that m6A modification is involved in various neurobiological processes such as learning and memory (Widagdo et al., 2016; Walters et al., 2017), neurogenesis (Li et al., 2017), synaptic plasticity (Merkurjev et al., 2018), and axonal growth (Yu et al., 2018). Recently, increasing attention has been focused on changes in the m6A methylation profile of nervous system diseases. A previous report showed that m6A modification levels in total RNA were decreased in the hippocampus after traumatic brain injury, which could be due to the downregulation of METTL3 (Wang et al., 2019). It has been reported that m6A levels in the cortex and hippocampus of Alzheimer’s disease (AD) mice were notably higher than those in the control group and that m6A methylation genes were involved in the pathways associated with the occurrence of AD (Han et al., 2020). Si et al. (2020) found that METTL3-mediated miRNA m6A methylation promotes stress granule formation and reduces the apoptosis of injury neurons in the early stage of acute IS. Furthermore, Wang et al. (2020a, b) found that the expression levels of METTL3 and WTAP were reduced in the larger pathological tissue of cerebral AVMs. Mechanistically, the lack of METTL3 reduced the level of heterodimeric Notch E3 ubiquitin ligase, thereby continuously activating the Notch signaling pathway, which affects the angiogenesis of endothelial cells. WTAP could regulate desmoplakin (DSP) expression through m6A modification, thereby affecting the angiogenesis of endothelial cells.

Although studies on neurological disorders and m6A modification have gradually increased in recent years, there is little research on the role of m6A in CIRI. In the present study, we detected the m6A levels in rat brain samples, which showed that the m6A levels in total RNA were significantly increased compared with the sham group. Meanwhile, there were no significant differences between the indicated time points in the MCAO/R groups. Therefore, we chose brain samples from the sham and MACO/R-24 groups for MeRIP-seq and RNA-seq. Further conjoint analyses of our MeRIP-seq and RNA-seq data identified 1,160 altered m6A methylated DEGs. Among them, 813 genes were significantly hypermethylated and upregulated (hyper-up), and 342 genes were hypomethylated and downregulated (hypo-down). For example, CAMKK2 (calcium/calmodulin-dependent protein kinase 2 (beta), a hypo-down DEG, is the positive AMPK regulator, which was found to be involved in autophagy inhibition (Chen et al., 2020). Chen et al. found that m6A modification affected AMPK activity by promoting the translation of the negative AMPK regulator PPM1A while decreasing the expression of CAMKK2 by reducing its mRNA stability, resulting in autophagy inhibition. CD14, a hyper-up DEG, plays an important role in the TLR-mediated immune response as a co-receptor for TLR2 and TLR4 (Yao et al., 2020). A previous study found that CD14 underwent more m6A modification in the MCAO/R rat brain, induced by a decrease in FTO. Combined with the results of the other two datasets (GSE78731 and GSE61616), we identified a total of 490 key genes for functional enrichment analysis. The KEGG pathway analysis showed that the upregulated key genes were mainly implicated in the pathway associated with immune and inflammatory responses. Similar to our research, Chokkalla et al. established a mouse model of transient ischemia and reperfusion to reveal the m6A transcriptome-wide map of CIRI and found that these mRNAs with altered m6A modifications were enriched in biological processes such as inflammation, apoptosis, and transcriptional regulation (Chokkalla et al., 2019). Diao et al. (2020) demonstrated that hypothermia could protect neurons from CIRI-induced proptosis *via* m6A-mediated activation of PTEN and reduce inflammatory injury by depressing the expression levels of the NLRP3 inflammasome and its related cytokines. In addition, the downregulated key genes were mainly implicated in the cAMP signaling pathway, neuroactive ligand–receptor interaction, and dopaminergic synapse. This suggests that the m6A modification in the brain cortex might be closely related to the damage of neurons and synapses, which provides new insight into the strategies for protecting neurons after CIRI.

As we know, m6A modification is dynamic and reversible, installed by the “writer,” removed by the “erasers,” and identified by the “reader” (Li et al., 2019). To investigate the main m6A modulator associated with elevated methylation levels in CIRI, we screened out differentially expressed m6A regulators between the sham and MCAO/R groups and validated by qRT-PCR and western blotting. Finally, FTO was identified. A reduction of FTO was thought to be associated with an increase in m6A methylation levels. Consistent with our results, a previous study (Xu et al., 2020) reported that the RNA m6A levels increased after both neuronal glucose oxygen deprivation/reoxygenation (OGD/R) treatment and rat MCAO/R treatment and that the demethylases, Alkbh5 and FTO, rather than the methylases, Mettl3 and Mettl14, were primarily responsible for abnormal m6A modification. Chokkalla et al. (2019) also found that m6A demethylase FTO mRNA expression decreased significantly at 3 to 24 h after MCAO/R surgery compared with sham. Notably, FTO is highly enriched in the brain, and its deficiency results in impaired dopaminergic neurotransmission and microcephaly (Hess et al., 2013). One study reported that FTO deficiency reduced anxiety and depression-like behaviors (Sun et al., 2019), and FTO-mediated m6A methylation might also be associated with Parkinson’s disease (Chen et al., 2019). In addition, we also observed a seemingly paradoxical increase in Alkbh5, a demethylase, after MCAO/R surgery compared with sham. This condition has also been reported in a previous study (Xu et al., 2020), which asserted that the increase in Alkbh5 expression likely compensates for the stress response in brain ischemia/hypoxia. Supporting this view, Mathiyalagan et al. (2019) found a compensatory increase in Alkbh5 expression around the infarct after heart failure.

To further investigate the cellular distribution of FTO, immunofluorescence staining was performed in the rat brain cortex. This showed that FTO is predominantly localized and specifically downregulated in neurons, consistent with a previous report (Chokkalla et al., 2019). Another study (Bai et al., 2018) also confirmed that FTO was present in the dopamine neurons of mice and that deficiency of FTO could impair immature neuronal differentiation in the subgranular zone of the hippocampus (Spychala and Ruther, 2019). However, the KEGG pathway analysis showed that hypomethylated DEGs were associated with neurons and nerve synapses, seemingly contradicting the distribution of decreased FTO. In the present study, we also detected an increase in Alkbh5 and hypothesized that it might be responsible for hypomethylated genes. Du et al. (2020) found that Alkbh5 was almost exclusively expressed in neurons but not in glial cells. In addition, downregulated FTO with some expression in Iba1+ cells might be responsible for hypermethylated genes enriched in the pathways related to immune and inflammatory responses. Next, we will adopt neurons and microglia to establish the OGD/R model, further study the alteration of Alkbh5 and FTO, and reveal the specific mechanisms of m6A demethylase in CIRI.

In addition, our study has several potential limitations. First, the amounts of rat sample we used for MeRIP-seq and RNA-seq are limited (three MCAO/R samples and three sham samples), which requires that we need to use the rat models to verify the results of bioinformatics analysis. In the present study, we just selected a few key m6A regulators for validation by qRT-PCR and western blotting. Furthermore, we found that reduction of FTO might be responsible for the increase of m6A methylation levels in MCAO/R rats, which was consistent with previous studies (Chokkalla et al., 2019; Xu et al., 2020). However, it is unclear whether regulating FTO expression could reduce neuronal death caused by CIRI. Based on these two potential limitations, in our next study, we will adopt cell models (oxygen-glucose deprivation and reoxygenation, OGD/R) and rat models (MCAO/R) to further explore the specific mechanism of FTO in cerebral ischemia–reperfusion injury.

## Conclusion

In conclusion, our present study characterized the differential m6A methylome in MCAO/R-operated rats relative to sham rats by analyzing the MeRIP-seq and RNA-seq data. This revealed the mechanism of alteration of m6A modification after CIRI, which might be primarily regulated by FTO. This study could be used as an initial roadmap for discovering m6A functions and suggests potential future research regarding m6A modifications in CIRI.

## Data Availability Statement

The RNA-seq data were deposited in the Gene Expression Omnibus database (Series# GSE163614, https://www.ncbi.nlm.nih.gov/geo/query/acc.cgi?acc=GSE163614). All datasets generated for this study are included in the article/ Supplementary Material or Gene Expression Omnibus database.

## Ethics Statement

The animal study was reviewed and approved by the Ethics Committee of the First Hospital of Jilin University (Changchun, China).

## Author Contributions

QL and XC initiated, coordinated, and drove the project. DY and QW performed the MCAO/R rat model and sequencing data analysis. YZ, YS, HY, and JW performed the RT-qPCR, western blot and immunofluorescence experiments. RL and ZS drafted the manuscript and the figures. QW critically revised the manuscript. All authors contributed to the article and approved the submitted version.

## Conflict of Interest

The authors declare that the research was conducted in the absence of any commercial or financial relationships that could be construed as a potential conflict of interest.

## Footnotes

1 http://www.ncbi.nlm.nih.gov/geo/

## Supplementary Material

Supplementary Figure 1 Pie chart showing the distribution of the differentially methylated m6A peaks in five non-overlapping segments.

Supplementary Figure 2 **(A,C)** The box plots of normalized data for the GSE61616 **(A)** and GSE78731 **(C)** datasets. **(B,D)** Volcano plots for the differentially expressed genes (DEGs) in the GSE61616 **(B)** and GSE78731 **(D)** datasets. The DEGs were identified with a *p*-value of < 0.05 and fold changes ≥ 2. The red plots represent the upregulated genes, and the blue plots represent the downregulated genes.

Supplementary Figure 3 Visualization of the top five hyper-up (hypo-down) DEGs in all three datasets ranged by fold change of the MeRIP-seq data. **(A)** The hyper-up DEGs include *Lgals3*, *C1qb*, *Itgb2*, *Hcls1*, and *Cd44*. **(B)** The hypo-down DEGs include *Pde10a*, *Camkk2*, *Gpr88*, *Neu2*, and *Drd2*. **(C)** qRT-PCR shows the mRNA expressions of *Lgals3, C1qb, Itgb2, Hcls1*, and *Cd44* in sham and MCAO/R groups. **(D)** qRT-PCR shows the mRNA expressions of *Pde10a, Camkk2, Gpr88, Neu2*, and *Drd2* in sham and MCAO/R groups. Data are presented as mean ± SD. ^∗∗^*p* < 0.01; ^∗∗∗^*p* < 0.001 vs. sham group, *n* = 5, student’s *t*-test.

Supplementary Table 1 Primers of top five hyper-up or hypo-down DEGs for qRT-PCR.

Supplementary Table 2 Detailed information on the 490 key genes.

Supplementary Table 3 Top 30 GO terms enriched by upregulated key genes.

Supplementary Table 4 Top 30 GO terms enriched by downregulated key genes.
