# Supplementary figures and images for: Alteration of N6 -Methyladenosine mRNA Methylation in a Rat Model of Cerebral Ischemia–Reperfusion Injury
Source: Front Neurosci. 2021 Mar 16;15:605654. doi: 10.3389/fnins.2021.605654 (PMC8009187; doi:10.3389/fnins.2021.605654)

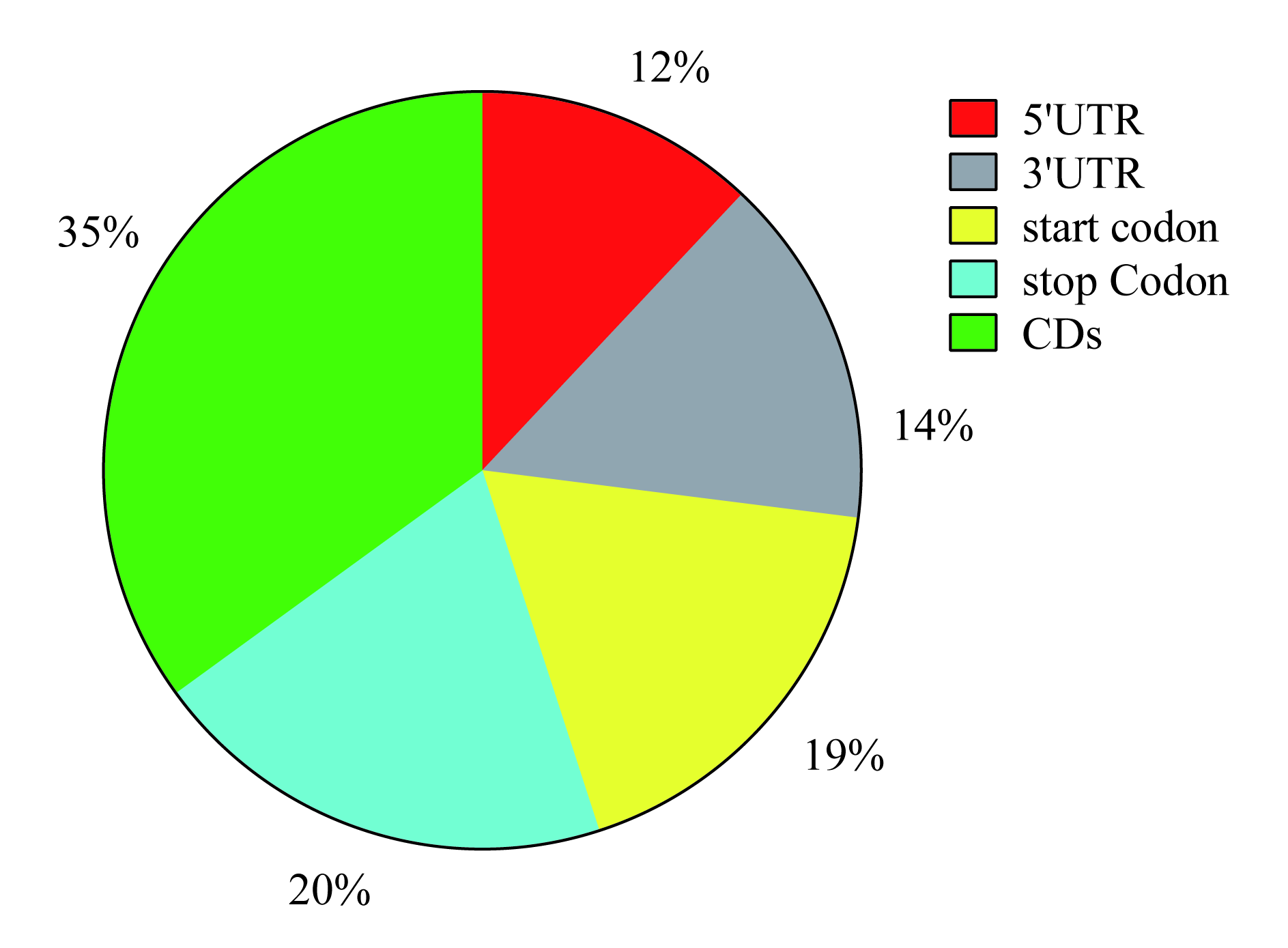

Supplement: Supplementary Figure 1 — Pie chart showing the distribution of the differentially methylated m6A peaks in five non-overlapping segments. [file Image_1.TIF]

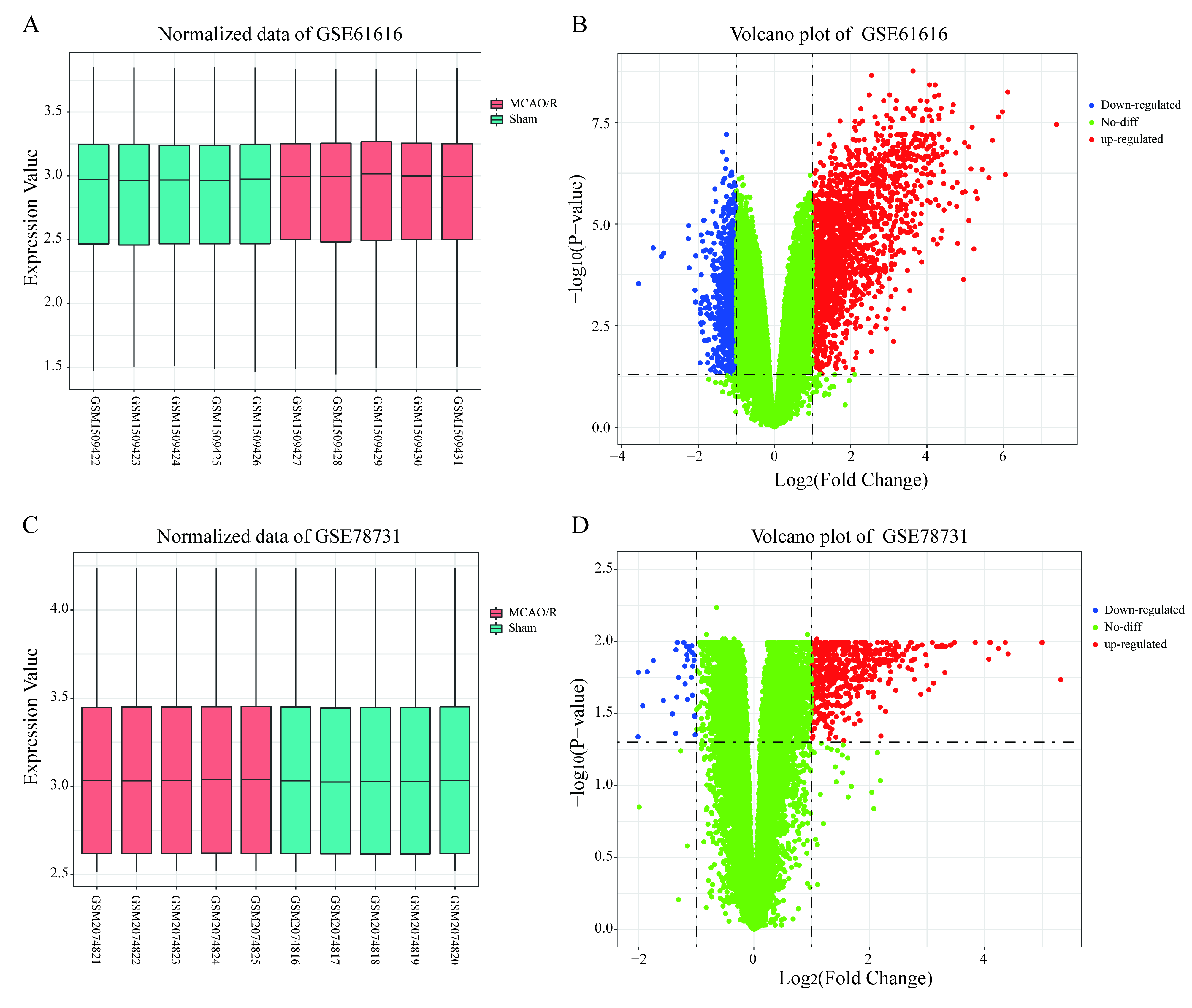

Supplement: Supplementary Figure 2 — (A,C) The box plots of normalized data for the GSE61616 (A) and GSE78731 (C) datasets. (B,D) Volcano plots for the differentially expressed genes (DEGs) in the GSE61616 (B) and GSE78731 (D) datasets. The DEGs were identified with a p-value of < 0.05 and fold changes ≥ 2. The red plots represent the upregulated genes, and the blue plots represent the downregulated genes. [file Image_2.TIF]

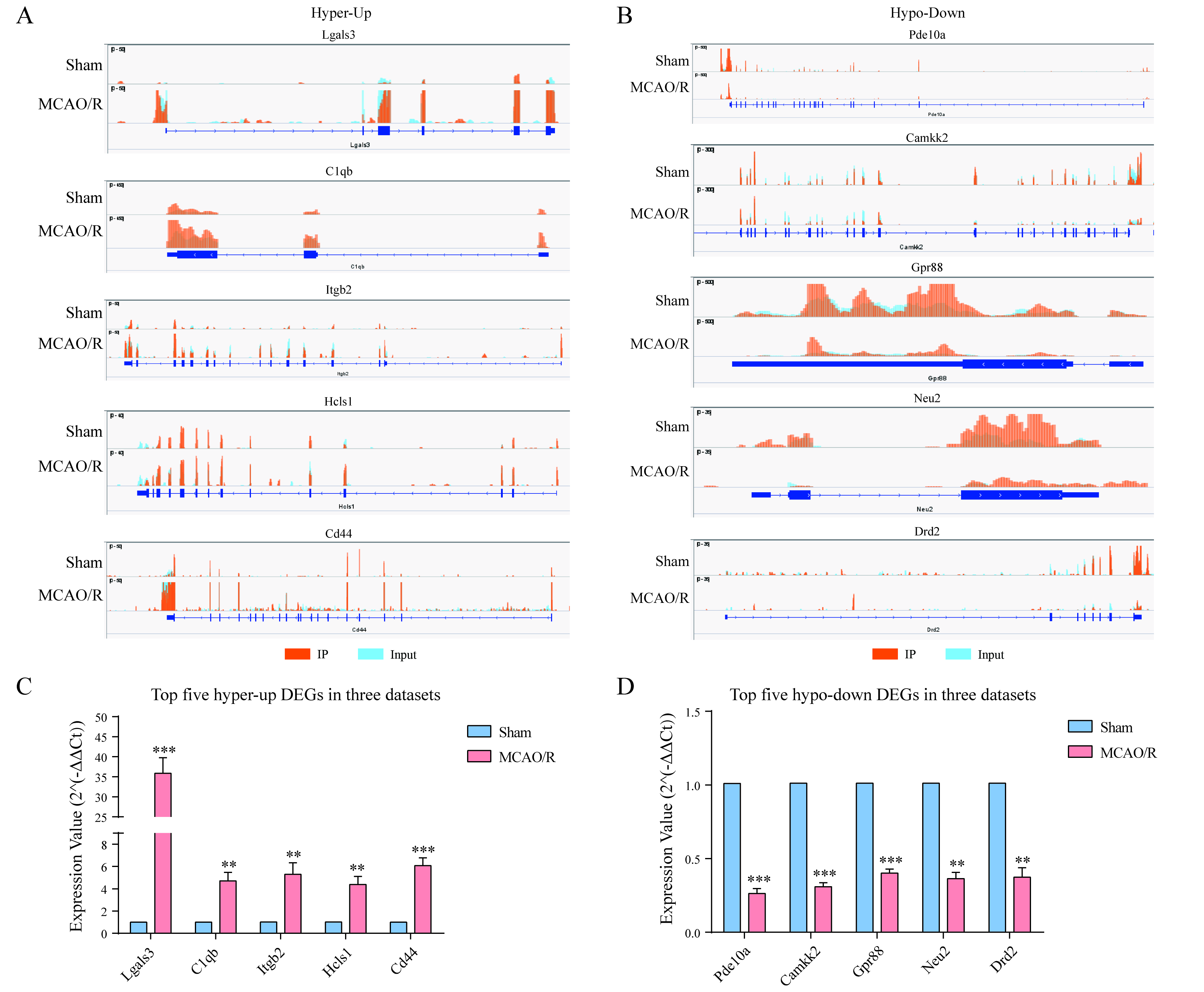

Supplement: Supplementary Figure 3 — Visualization of the top five hyper-up (hypo-down) DEGs in all three datasets ranged by fold change of the MeRIP-seq data. (A) The hyper-up DEGs include Lgals3, C1qb, Itgb2, Hcls1, and Cd44. (B) The hypo-down DEGs include Pde10a, Camkk2, Gpr88, Neu2, and Drd2. (C) qRT-PCR shows the mRNA expressions of Lgals3, C1qb, Itgb2, Hcls1, and Cd44 in sham and MCAO/R groups. (D) qRT-PCR shows the mRNA expressions of Pde10a, Camkk2, Gpr88, Neu2, and Drd2 in sham and MCAO/R groups. Data are presented as mean ± SD. ∗∗p < 0.01; ∗∗∗p < 0.001 vs. sham group, n = 5, student’s t-test. [file Image_3.TIF]
